# Supplementary figures and images for: SNTA1 gene rescues ion channel function and is antiarrhythmic in cardiomyocytes derived from induced pluripotent stem cells from muscular dystrophy patients
Source: eLife. 2022 Jun 28;11:e76576. doi: 10.7554/eLife.76576 (PMC9239678; doi:10.7554/eLife.76576)

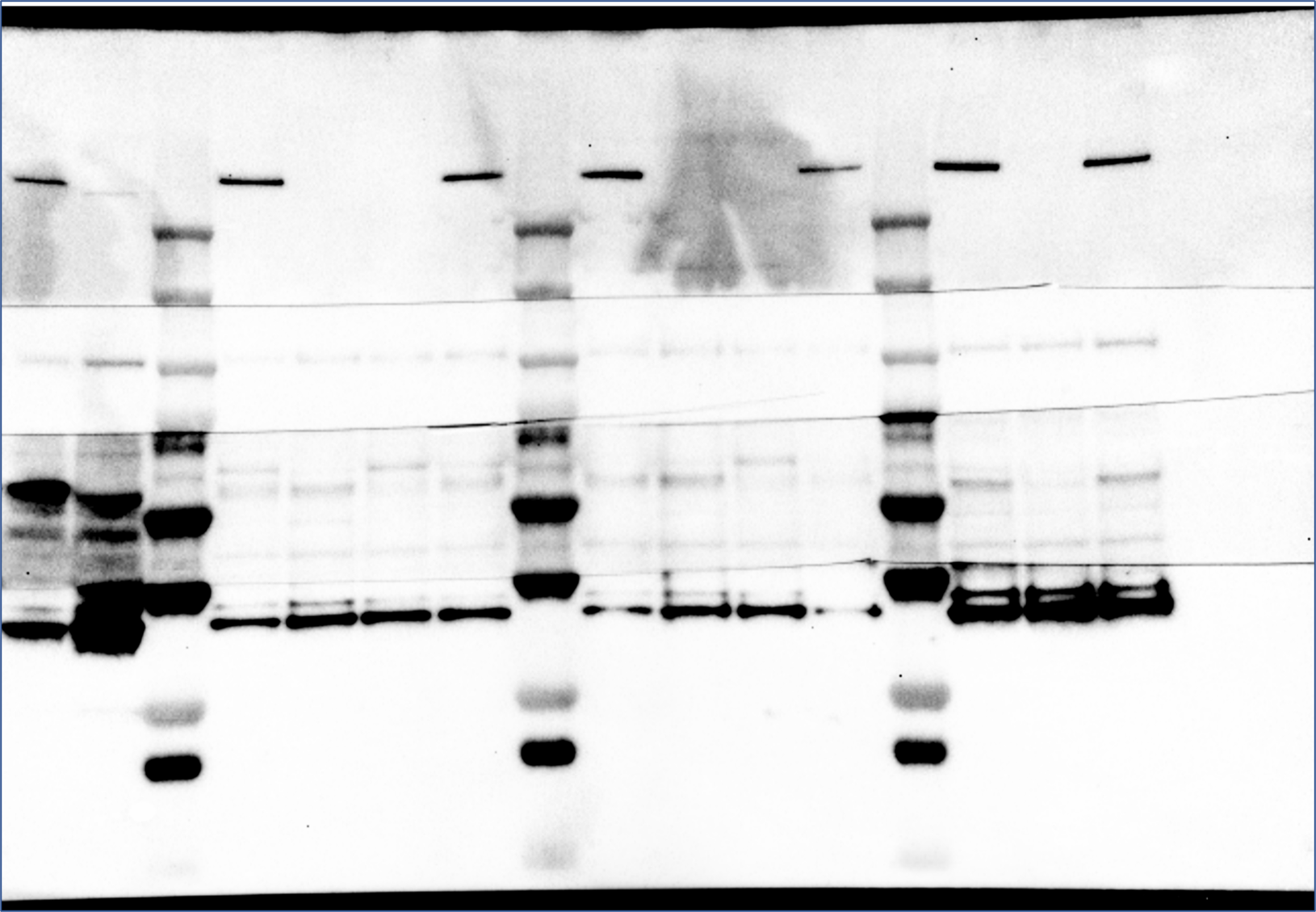

Supplement: Figure 2—source data 1. [file elife-76576-fig2-data1.zip › Figure 2-source data 1/Figure 2-source data 1. Full unedited gel_Fig 2A.tif]

Full unedited gel for  
Figure 2A

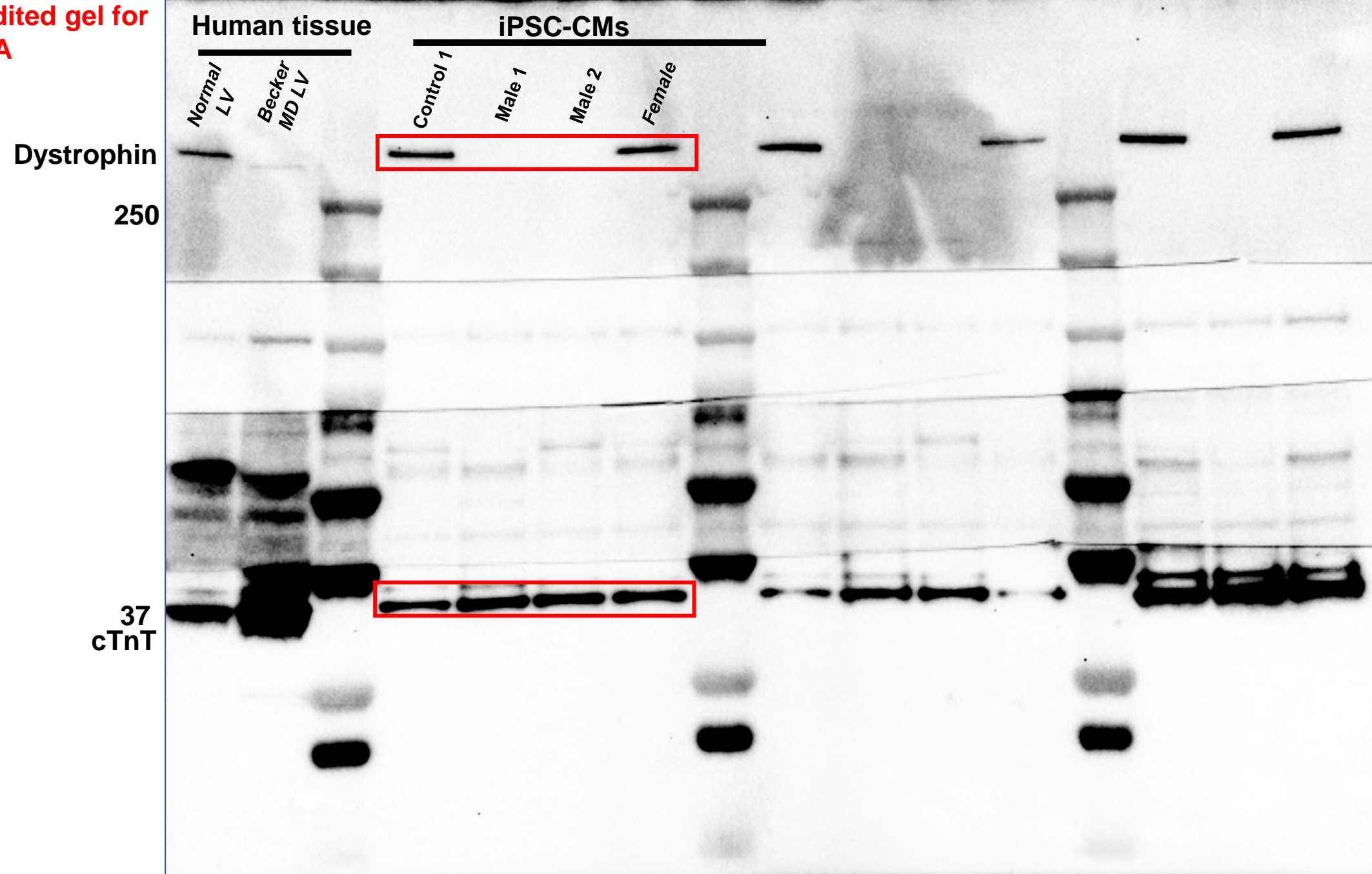

Supplement: Figure 2—source data 1. [file elife-76576-fig2-data1.zip › Figure 2-source data 1/Figure 2-source data 2. Unedited gel for figures 2A.pdf]

Full unedited gel for  
Supplementary  
Figure 4A

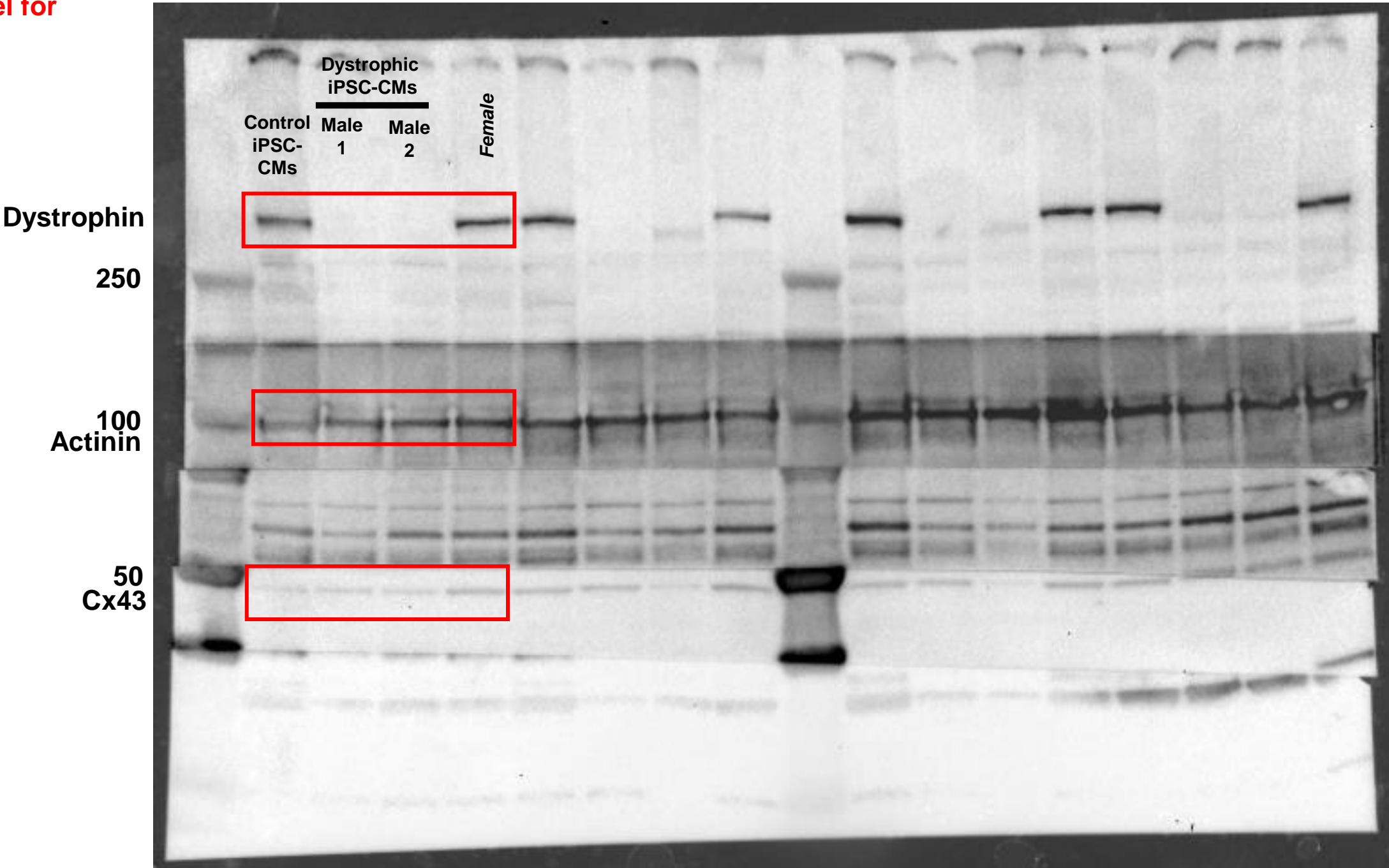

Supplement: Figure 4—figure supplement 2—source data 1. [file elife-76576-fig4-figsupp2-data1.zip › Figure 4-figure supplement 2-source data 1/Supplemental Figure 4-source data 2. Unedited gel for Supplementary Figure 4A.pdf]

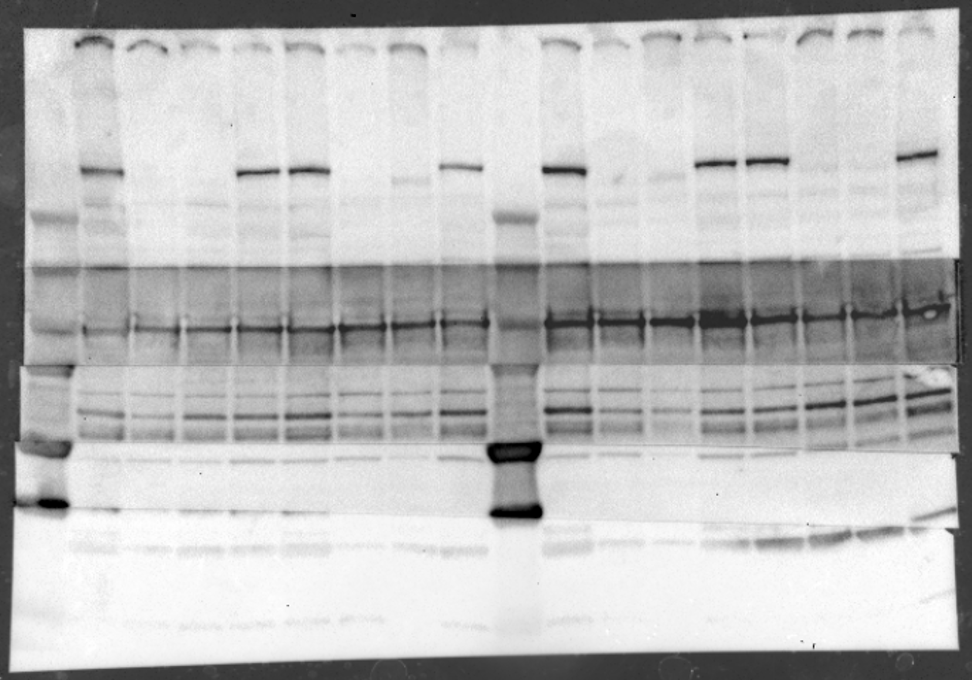

Supplement: Figure 4—figure supplement 2—source data 1. [file elife-76576-fig4-figsupp2-data1.zip › Figure 4-figure supplement 2-source data 1/Supplemental Figure 4-source data 1. Full unedited gel_Suppl. Fig 4A.tif]

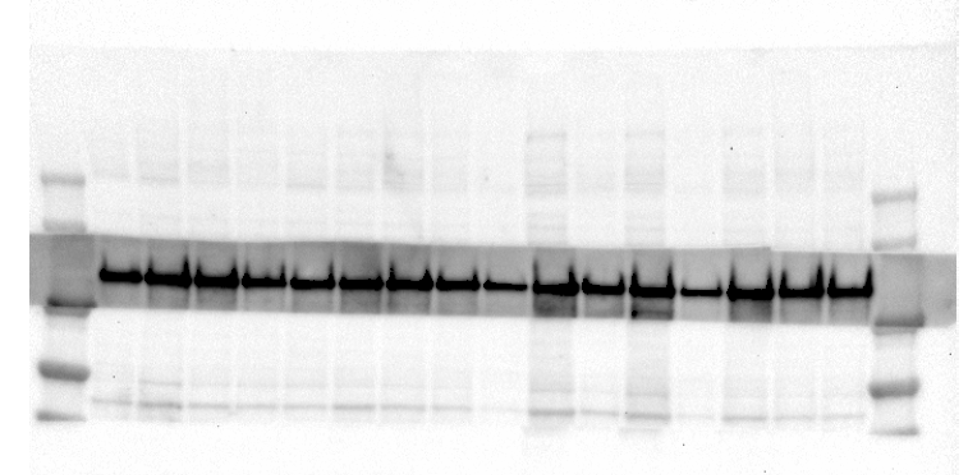

Supplement: Figure 5—figure supplement 4—source data 1. [file elife-76576-fig5-figsupp4-data1.zip › Figure 5-figure supplement 4-source data 1/Figure 5-figure supplement 4-source data 1. Full unedited gel_Suppl. Fig 5supplA.tif]

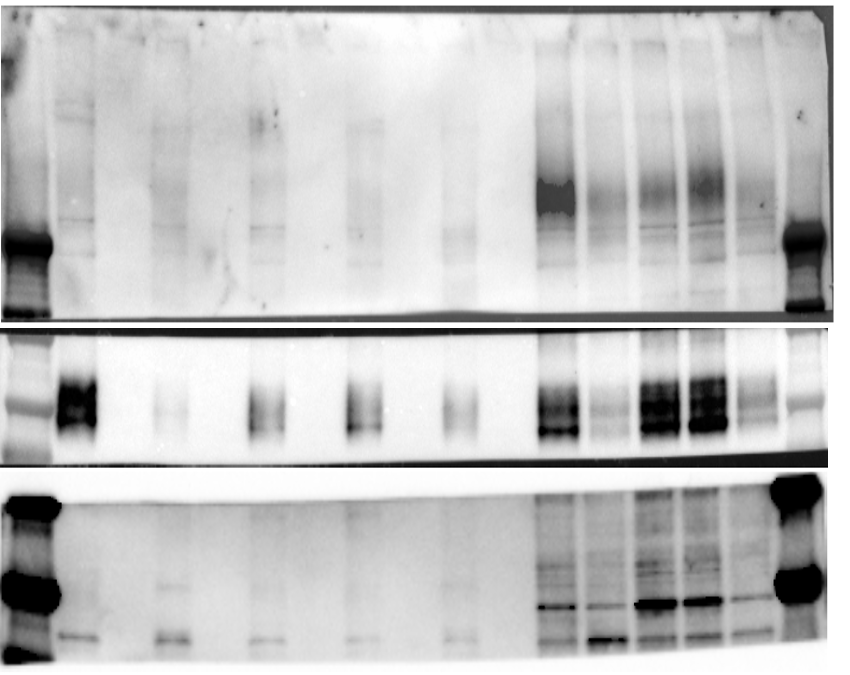

Supplement: Figure 5—figure supplement 4—source data 1. [file elife-76576-fig5-figsupp4-data1.zip › Figure 5-figure supplement 4-source data 1/Figure 5-figure supplement 4-source data 2. Full unedited gel_Suppl. Fig 8C.tif]
