# Supplementary material for: SNTA1 gene rescues ion channel function and is antiarrhythmic in cardiomyocytes derived from induced pluripotent stem cells from muscular dystrophy patients
Source: eLife. 2022 Jun 28;11:e76576. doi: 10.7554/eLife.76576 (PMC9239678; doi:10.7554/eLife.76576)

Full unedited gel for Figure 5 - Figure supplement 4 - a

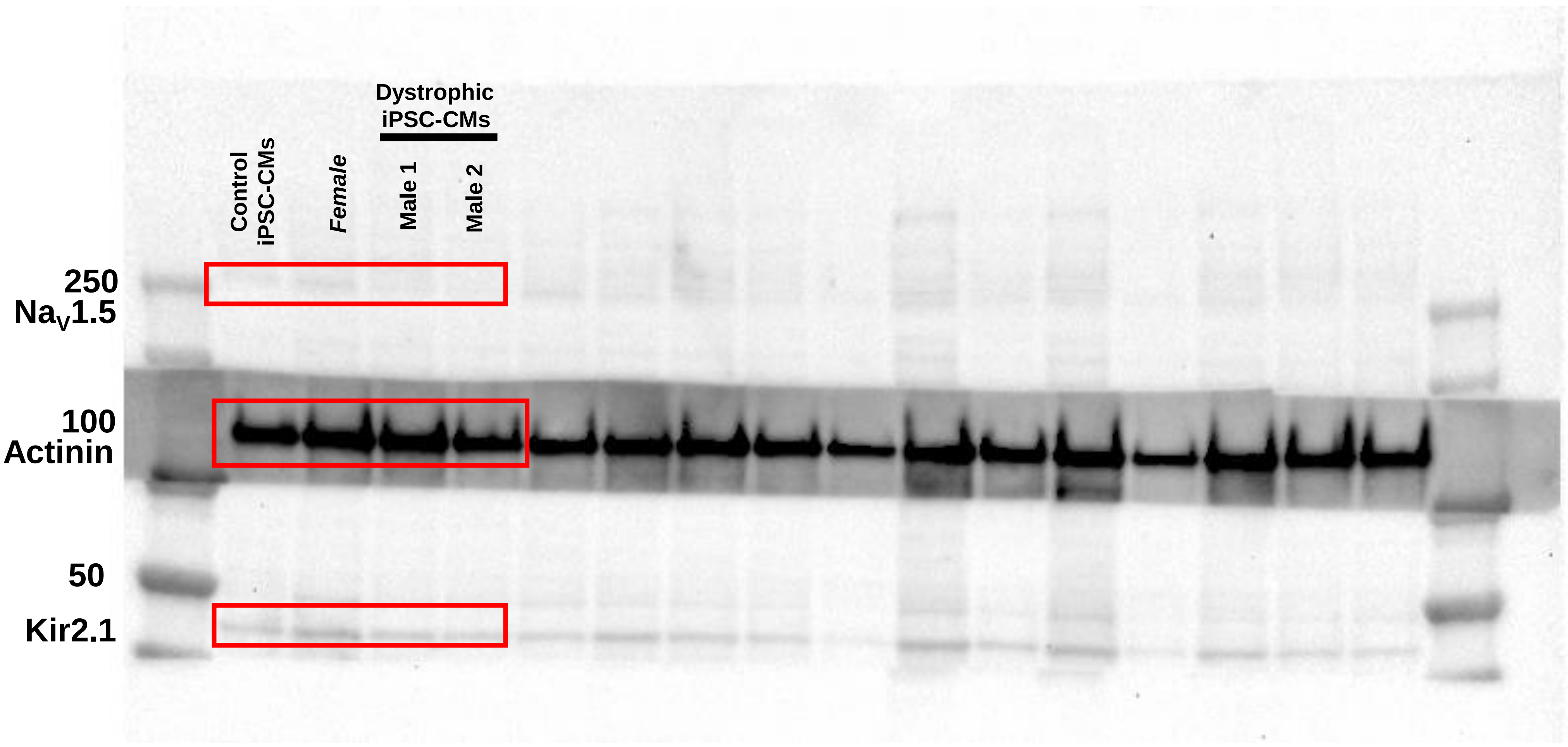

Full unedited gel for Figure 5 - Figure supplement 4 - c

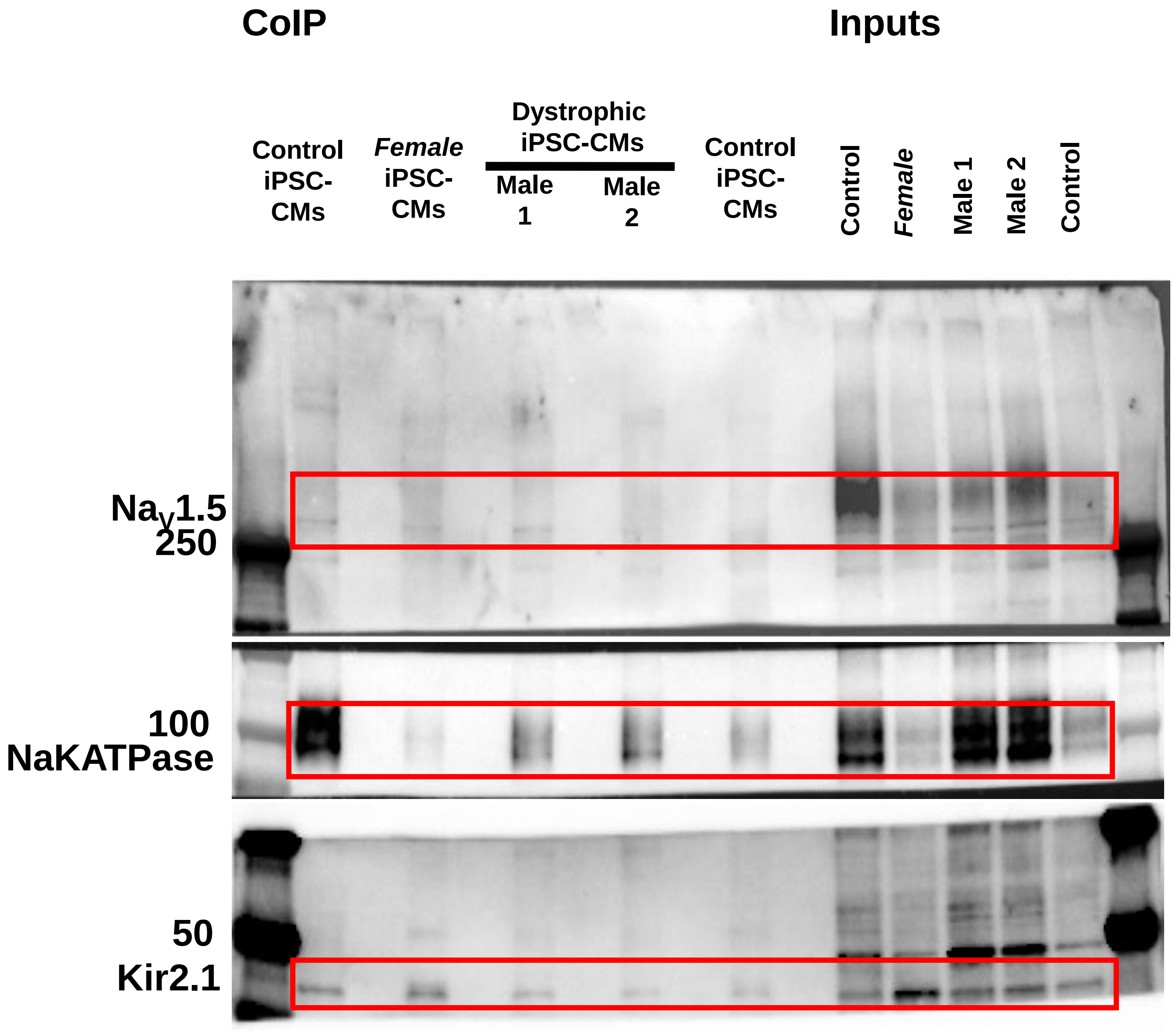

Supplement: Figure 5—figure supplement 4—source data 1. [file elife-76576-fig5-figsupp4-data1.zip › Figure 5-figure supplement 4-source data 1/Figure 5-figure supplement 4-source data.pdf]
