# Supplementary material for: SNTA1 gene rescues ion channel function and is antiarrhythmic in cardiomyocytes derived from induced pluripotent stem cells from muscular dystrophy patients
Source: eLife. 2022 Jun 28;11:e76576. doi: 10.7554/eLife.76576 (PMC9239678; doi:10.7554/eLife.76576)
Supplement: Supplementary file 1. [file elife-76576-supp1.docx]

**Supplementary File 1 (Table 1)** Action potential parameters of iPSC-CMs vs Control 1, all paced at 1 or 2 Hz.

| **Group** | **dV/dt_max_** | **Overshoot** | **Amplitude** | **MDP** | **APD_90_** | ***n*** |
| --- | --- | --- | --- | --- | --- | --- |
| **1 Hz** |  |  |  |  |  |  |
| Control 1 | 32 ± 5 | 31 ± 2 | 101 ± 3 | -70 ± 2 | 171 ± 17 | 13 |
| Male 2 | 8 ± 1* | 23 ± 2* | 85 ± 2* | -64 ± 2 | 171 ± 19 | 12 |
| Male 1 | 11 ± 1* | 31 ± 1 | 103 ± 2 | -70 ± 2 | 218 ± 21 | 15 |
| Female | 12 ± 2 | 28 ± 3 | 92 ± 4 | -63 ± 2* | 169 ± 22 | 9 |
| **2 Hz** |  |  |  |  |  |  |
| Control 1 | 39 ± 9 | 32 ± 2 | 102 ± 3 | -70 ± 1 | 162 ± 12 | 15 |
| Male 2 | 11 ± 2**** | 27 ± 2 | 92 ± 3* | -65 ± 1* | 171 ± 14 | 16 |
| Male 1 | 11 ± 1**** | 29 ± 1 | 99 ± 3 | -70 ± 2 | 186 ± 16 | 17 |
| Female | 9 ± 1*** | 28 ± 4 | 91 ± 5 | -63 ± 2** | 149 ± 16 | 7 |

One-way ANOVA followed by Dunnett’s multiple comparisons test. Values are expressed as mean ± SEM. *****P* < 0.0001, ****P* = 0.0002, ***P* = 0.0081, and **P* < 0.05
