# Supplementary material for: SNTA1 gene rescues ion channel function and is antiarrhythmic in cardiomyocytes derived from induced pluripotent stem cells from muscular dystrophy patients
Source: eLife. 2022 Jun 28;11:e76576. doi: 10.7554/eLife.76576 (PMC9239678; doi:10.7554/eLife.76576)
Supplement: Supplementary file 2. [file elife-76576-supp2.docx]

**Supplementary File 2 (Table 2).** Action potential parameters of iPSC-CMs vs Control 2, all paced at 1 or Hz.

| **Group** | **dV/dt_max_** | **Overshoot** | **Amplitude** | **MDP** | **APD_90_** | ***n*** |
| --- | --- | --- | --- | --- | --- | --- |
| **1 Hz** |  |  |  |  |  |  |
| Control 2 | 66 ± 12 | 38 ± 2 | 106 ± 2 | -69 ± 2 | 204 ± 18 | 14 |
| Male 2 | 9.6 ± 2*** | 23 ± 2**** | 88 ± 3*** | -64 ± 2 | 171 ± 19 | 12 |
| Male 1 | 11 ± 1**** | 32 ± 1 | 103 ± 2 | -70 ± 2 | 218 ± 21 | 15 |
| Female | 12 ± 2*** | 28 ± 3* | 92 ± 4** | -63 ± 2 | 169 ± 22 | 9 |
| **2 Hz** |  |  |  |  |  |  |
| Control 2 | 58 ± 11 | 39 ± 2 | 102 ± 2 | -67 ± 1 | 150 ± 9 | 14 |
| Male 2 | 11 ± 2**** | 27 ± 2** | 92 ± 3 | -65 ± 1 | 171 ± 14 | 16 |
| Male 1 | 11 ± 1**** | 29 ± 1** | 99 ± 3 | -70 ± 2 | 186 ± 16 | 17 |
| Female | 9 ± 1*** | 28 ± 4* | 91 ± 5 | -63 ± 2 | 149 ± 16 | 7 |

One-way ANOVA followed by Dunnett’s multiple comparisons test. Values are expressed as mean ± SEM. *****P* < 0.0001, ****P* < 0.0007, ***P* < 0.0089, and **P* < 0.05.
