# Supplementary material for: SNTA1 gene rescues ion channel function and is antiarrhythmic in cardiomyocytes derived from induced pluripotent stem cells from muscular dystrophy patients
Source: eLife. 2022 Jun 28;11:e76576. doi: 10.7554/eLife.76576 (PMC9239678; doi:10.7554/eLife.76576)
Supplement: Supplementary file 3. [file elife-76576-supp3.docx]

**Supplementary File 3 (Table 3)** Action potential parameters of iPSC-CMs at 1 and 2 Hz, Control 1 vs Control 2.

| **Group** | **dV/dt_max_** | **Overshoot** | **Amplitude** | **MDP** | **APD_90_** | ***n*** |
| --- | --- | --- | --- | --- | --- | --- |
| **1 Hz** |  |  |  |  |  |  |
| Control 1 | 44 ± 13 | 31 ± 2 | 101 ± 3 | -70 ± 2 | 171 ± 17 | 13 |
| Control 2 | 66 ± 12 | 38 ± 2 | 106 ± 2 | -69 ± 2 | 204 ± 18 | 14 |
| **2 Hz** |  |  |  |  |  |  |
| Control 1 | 39 ± 9 | 32 ± 2 | 102 ± 3 | -70 ± 1 | 162 ± 12 | 15 |
| Control 2 | 58 ± 11 | 39 ± 2 | 102 ± 2 | -67 ± 1 | 150 ± 9 | 14 |

One-way ANOVA followed by Dunnett’s multiple comparisons test. Values are expressed as mean ± SEM.
