# Supplementary material for: SNTA1 gene rescues ion channel function and is antiarrhythmic in cardiomyocytes derived from induced pluripotent stem cells from muscular dystrophy patients
Source: eLife. 2022 Jun 28;11:e76576. doi: 10.7554/eLife.76576 (PMC9239678; doi:10.7554/eLife.76576)
Supplement: Supplementary file 5. [file elife-76576-supp5.docx]

**Supplementary File 5 (Table 5).** Biophysical parameters of DMD, and female iPSC-CMs vs Control 2.

|  | | **Activation** | |  | | |  | |
| --- | --- | --- | --- | --- | --- | --- | --- | --- |
|  | **V_50_** | | ***k*** | | **V_rev_** | **Peak current density** | | ***n*** |
| **Na^+^ currents** | mV | | mV | | mV | pA/pF | |  |
| Control 2  Male 1  Male 2  Female | -34 ± 1  -31 ± 1  -28 ± 1  -27 ± 1 | | 4 ± 1  4 ± 1  3 ± 1  4 ± 1 | | 15 ± 1  16 ± 1  17 ± 2  14 ± 1 | -38 ± 1  -13 ± 1****  -15 ± 1****  -11 ± 1**** | | 15  11  9  22 |
| **Ca^2+^ currents** |  | | | | | | | |
| Control 2  Male 1  Male 2  Female | -11 ± 1  -16 ± 1  -14 ± 1  -12 ± 1 | | 8 ± 1  8 ± 1  9 ± 1  7 ± 1 | | 55 ± 1  54 ± 2  57 ± 1  54 ± 2 | -10 ± 1  -9 ± 1  -9 ± 1  -10 ± 1 | | 11  14  10  13 |

Activation parameters were calculated by data fitting to Boltzmann functions. V_50_ is the voltage for half‑maximal activation, *k* is the slope factor and *n* the number of cells. One-way ANOVA followed by Dunnett’s multiple comparisons test. Values are expressed as mean ± s.e.m. *****P* < 0.0001.
