# Supplementary material for: SNTA1 gene rescues ion channel function and is antiarrhythmic in cardiomyocytes derived from induced pluripotent stem cells from muscular dystrophy patients
Source: eLife. 2022 Jun 28;11:e76576. doi: 10.7554/eLife.76576 (PMC9239678; doi:10.7554/eLife.76576)
Supplement: Supplementary file 6. [file elife-76576-supp6.docx]

**Supplementary File 6 (Table 6).** Biophysical parameters of iPSC-CMs, Control 1 vs Control 2

|  | | **Activation** | |  | | |  | |
| --- | --- | --- | --- | --- | --- | --- | --- | --- |
|  | **V_50_** | | ***k*** | | **V_rev_** | **Peak current density** | | ***n*** |
| **Na^+^ currents** | mV | | mV | | mV | pA/pF | |  |
| Control 1  Control 2 | -29 ± 1  -34 ± 1 | | 3 ± 1  4 ± 1 | | 17 ± 1  15 ± 1 | -27 ± 1*  -38 ± 1 | | 14  15 |
| **Ca^2+^ currents** |  | | | | | | | |
| Control 1  Control 2 | - 8 ±1  -11± 1 | | 8 ± 1  8 ± 1 | | 54 ± 1  55 ± 2 | -10 ± 1  -10 ± 1 | | 9  11 |

Activation parameters were calculated by data fitting to Boltzmann functions. V_50_ is the voltage for half‑maximal activation, *k* is the slope factor and *n* the number of cells. One-way ANOVA followed by Dunnett’s multiple comparisons test. Values are expressed as mean ± s.e.m. **P* < 0.05.
