# Supplementary material for: SNTA1 gene rescues ion channel function and is antiarrhythmic in cardiomyocytes derived from induced pluripotent stem cells from muscular dystrophy patients
Source: eLife. 2022 Jun 28;11:e76576. doi: 10.7554/eLife.76576 (PMC9239678; doi:10.7554/eLife.76576)
Supplement: Supplementary file 7. [file elife-76576-supp7.docx]

**Supplementary File 7 (Table 7)** Primers used in mRNA analysis

| SCN5A (cDNA) | 5’-GAGGACCTGGACCCCTTCTA-3’ (forward primer)  5’-GCATGTTGAAGAGCGAGTGA-3’ (reverse primer |
| --- | --- |
| CACNA1C (cDNA) | 5’-AAGGCTACCTGGATTGGATCAC-3’ (forward primer)  5’-GCCACGTTTTCGGTGTTGAC-3’ (reverse primer) |
| KCNJ2 (cDNA) | 5’-TCCGTGACATCTGAAACCA-3’ (forward primer)  5’-TCACGGCTGCCTTCCTCTT-3’ (reverse primer) |
| 18s internal control (cDNA) | 5’-AACTTTCGATGGTAGTCGCCGT-3’ (forward primer)  5’-TCCTTGGATGTGGTAGCCGTTT-3’ (reverse primer) |
